# Enterprising spirit and college students’ creativity: the mediating role of growth mindset, creative tendency and the moderating role of cultural tightness

**DOI:** 10.3389/fpsyg.2025.1628948

**Published:** 2025-10-22

**Authors:** Guizhi Ma, Silin Zhou, Jiaqin Yang, Xiaotong Man, Chunlei Liu

**Affiliations:** ^1^Faculty of Education, Qufu Normal University, Qufu, China; ^2^School of Psychology, Qufu Normal University, Qufu, China

**Keywords:** enterprising spirit, creativity, cultural tightness, growth mindset, creative tendency

## Abstract

In an era of innovation-driven development, understanding how to cultivate creativity among college students is crucial. Enterprising spirit, as a key internal driver, is hypothesized to significantly promote creativity, yet its underlying mechanisms and boundary conditions remain underexplored. This study investigates the relationship between enterprising spirit and creativity among 522 college students, exploring the mediating roles of growth mindset and creative tendency and the moderating effect of cultural tightness. Data were collected using validated scales, including the college students’ enterprising spirit questionnaire, the Williams creativity tendency test scale, the cultural tightness–looseness scale, and measures of incremental/radical creativity and growth mindset. Descriptive statistics were conducted utilizing SPSS 26.0, Mediation and moderation effects were analyzed using PROCESS macro v4.0, and correlation network analysis was conducted using R software. The results showed that: (1) Enterprising spirit significantly predicted both incremental and radical creativity. (2) Growth mindset and creative tendency partially mediated this relationship. The indirect effect through growth mindset was significant (3) Cultural tightness moderated the second stage of the mediation path via creative tendency. The moderating effect analysis revealed this indirect effect was stronger in tight cultural environments. These findings reveal a complex mechanism through which enterprising spirit influences creativity, highlighting the interplay between individual factors and socio-cultural context. This study offers theoretical insights for positive psychology and provides practical implications for fostering creativity in higher education.

## 1 Introduction

Creativity, defined as the ability to produce novel and useful ideas or products, is universally recognized as a critical driver of scientific advancement, economic growth, and societal well-being (Runco and Acar, 2012). In the context of higher education, fostering creativity in college students is paramount, as they are the future innovators who will address complex global challenges. However, despite its recognized importance, researchers continue to debate the best pathways to cultivate this essential skill, particularly in non-Western contexts (Karami et al., 2024; Tang et al., 2023).

While numerous individual traits have been linked to creative performance, one promising yet underexplored antecedent is enterprising spirit—a disposition characterized by proactive goal-setting, resilience in the face of obstacles, and a persistent drive for self-actualization (Li and Huang, 2022). Rooted in both contemporary psychology and Confucian philosophy, this construct captures a powerful motivational force that may propel students to engage in and persist with creative tasks. Existing literature suggests a positive correlation between enterprising spirit and related outcomes like innovation capability (Zhang et al., 2014) and achievement motivation (Chen et al., 2020). However, a direct empirical investigation into its relationship with creativity, and the mechanisms underlying this relationship, is lacking.

This study proposes that enterprising spirit influences creativity through two distinct psychological pathways. First, we hypothesize that a growth mindset (Dweck, 2006) acts as a core mediating belief. Individuals with an enterprising spirit, characterized by hope and self-confidence, are likely to believe their abilities can be developed (Dweck, 2006). This mindset fosters a focus on learning and persistence after setbacks, which is essential for the iterative creative process (Royston and Reiter-Palmon, 2019). Second, we posit that creative tendency—a personality trait encompassing risk-taking and exploration (Guilford, 1950)—serves as another mediator. The courageous and adventurous dimensions of enterprising spirit may directly manifest as a stronger tendency to engage in creative behaviors.

Furthermore, the strength of these mechanisms may depend on the broader cultural context. According to cultural tightness–looseness theory (Gelfand et al., 2011), societies differ in the strength of their social norms and tolerance for deviance. We propose that cultural tightness moderates the efficacy of the second pathway. In tight cultures with strong norms, the clear expectations and high collective efficacy may provide a structured environment that channels a pre-existing creative tendency into tangible outcomes, whereas in loose cultures, the same tendency might dissipate without clear guidance (Chua et al., 2015; Zhang, 2022). This aligns with the Culture Fit theory (Lu et al., 2024), which suggests individuals thrive when their predispositions align with cultural expectations.

Therefore, to address these theoretical gaps, the present study constructs a moderated mediation model. We aim to investigate: (1) the direct effect of enterprising spirit on college students’ creativity; (2) the mediating roles of growth mindset and creative tendency in this relationship; and (3) the moderating role of cultural tightness on the path from creative tendency to creativity. To explore the complex interplay between these variables beyond traditional regression, we will also employ correlation network analysis to provide a holistic visualization of their relationships. This comprehensive approach seeks to offer a more nuanced understanding of how personal drive is translated into creative achievement within specific cultural environments, thereby contributing valuable insights to the fields of educational psychology and creativity research.

## 2 Literature review and hypotheses development

### 2.1 Enterprising spirit and creativity

Creativity, the capacity to generate ideas or products that are both novel and useful, is a cornerstone of innovation and problem-solving (Sternberg and Lubart, 1996; Runco and Acar, 2012). In educational contexts, fostering creativity is essential for preparing students to navigate complex, real-world challenges (Karami et al., 2024). Contemporary research often distinguishes between radical creativity (groundbreaking, paradigm-shifting ideas) and incremental creativity (refinements and extensions of existing ideas), acknowledging that different contexts may value each type differently (Madjar et al., 2011; Zhang, 2022).

A key antecedent to creative achievement is motivation. While previous studies have focused on constructs like achievement motivation (Chen et al., 2020), the role of enterprising spirit remains underexplored in international literature. Rooted in both Confucian philosophy and modern positive psychology, enterprising spirit is defined as an endogenous drive that enables individuals to set meaningful goals, believe in their potential to achieve them, and persevere through challenges with proactive and resilient behaviors (Li and Huang, 2022). It encompasses dimensions such as hope, self-confidence, courage, and perseverance.

Theoretically, enterprising spirit should be a powerful predictor of creativity. The goal-directed persistence and resilience it embodies are critical for navigating the inherent uncertainties and setbacks of the creative process (Dweck, 2006). Individuals high in enterprising spirit are more likely to view creative challenges as opportunities rather than threats, driving them to invest the necessary effort to generate and develop novel ideas (Kim et al., 2010). Empirical evidence supports this link, showing that enterprising spirit is correlated with related outcomes like innovation capability (Zhang et al., 2014). We therefore hypothesize:

*H1*: Enterprising spirit is positively related to creativity, such that higher levels of enterprising spirit predict higher levels of both (a) incremental and (b) radical creativity.

### 2.2 The mediating role of growth mindset

To understand how enterprising spirit boosts creativity, we examine growth mindset as a key mediating mechanism. Growth mindset, a concept from Dweck’s (1986, 2006) implicit theories, refers to the belief that one’s abilities and intelligence can be developed through dedication and effort.

We propose that enterprising spirit fosters this adaptive belief system. The “hope” and “self-confidence” dimensions of enterprising spirit are conceptually aligned with the core tenets of a growth mindset; individuals who are enterprising inherently believe in their capacity to grow and overcome obstacles. This mindset, in turn, is a known catalyst for creativity. Individuals with a growth mindset are more likely to embrace challenging tasks, persist in the face of failure, and view effort as a path to mastery—all essential for creative endeavor (Royston and Reiter-Palmon, 2019; King et al., 2012). They experience more positive affect during creative tasks, which enhances cognitive flexibility and original thinking (Nijstad et al., 2010; Gardner et al., 2015). Thus, an enterprising spirit may enhance creativity by cultivating the core belief that one can grow and improve.

*H2*: Growth mindset mediates the relationship between enterprising spirit and creativity.

### 2.3 The mediating role of creative tendency

Beyond belief systems, we posit that enterprising spirit also operates through a personality pathway, namely creative tendency (also called creative personality). This construct refers to a stable predisposition to engage in creative behaviors, characterized by traits like risk-taking, curiosity, and intellectual openness (Guilford, 1950; Barron and Harrington, 1981).

The “courage” dimension of enterprising spirit—which involves taking initiative and daring to be different—likely manifests as a stronger creative tendency. Enterprising individuals are more adventurous and less afraid of the social risks associated of proposing novel, non-conformist ideas. This tendency then directly translates into more frequent creative thought and action (Sternberg and Lubart, 1996). Therefore, we propose a second mediating mechanism:

*H3*: Creative tendency mediates the relationship between enterprising spirit and creativity.

### 2.4 The moderating role of cultural tightness

The translation of personal drive into creative output does not occur in a vacuum; it is shaped by the broader socio-cultural context (Zhang et al., 2016). Cultural tightness–looseness is a critical cultural dimension that describes the strength of social norms and the severity of sanctions for deviating from them (Gelfand et al., 2011). Tight cultures have strong, pervasive norms and low tolerance for deviance, while loose cultures have weaker norms and greater permissiveness.

We theorize that cultural tightness acts as a boundary condition, particularly for the personality pathway (*H3*). According to Culture Fit Theory (Lu et al., 2024), individuals are motivated to pursue behaviors that are consistent with cultural values and expectations. In a tight culture, clear norms and high collective efficacy can provide a structured environment that productively channels a pre-existing creative tendency. The high sense of order and shared purpose may help individuals focus their creative efforts on goals that are socially validated, thereby increasing the likelihood of tangible creative outcomes, particularly of the incremental kind (Cremer and Loebbecke, 2020; Zhang, 2022). Conversely, in loose cultures, the lack of clear structure might lead a strong creative tendency to dissipate without a clear focus.

However, we do not expect cultural tightness to moderate the growth mindset pathway (*H2*). A growth mindset is an internal, stable belief about the malleability of self. Its effect on motivating effort and persistence towards creative goals is likely to be robust across different cultural environments, as it operates primarily at the individual psychological level.

*H4*: Cultural tightness moderates the second stage of the mediation path via creative tendency. The positive relationship between creative tendency and creativity is stronger for individuals in tight cultures than for those in loose cultures.

### 2.5 The present research and theoretical model

**Figure 1:**
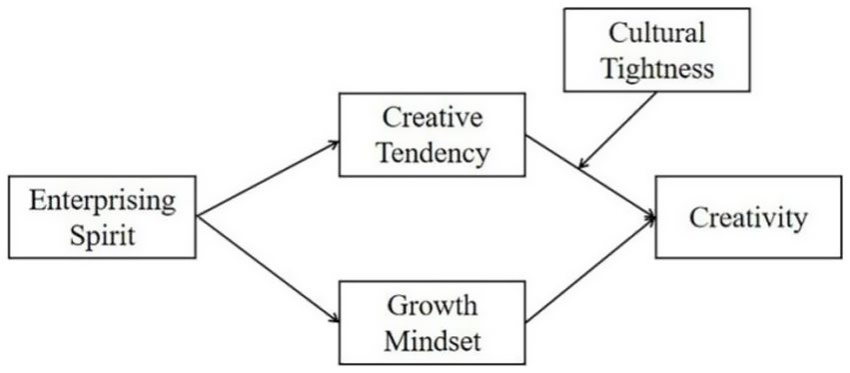
The proposed moderated mediation model.

In summary, this study proposes a moderated mediation model (see Figure 1) to elucidate the mechanisms and boundary conditions linking enterprising spirit to creativity. We hypothesize that enterprising spirit influences creativity through two distinct pathways: (1) by fostering a growth mindset and (2) by enhancing creative tendency. Furthermore, we posit that the effectiveness of the creative tendency pathway is enhanced in tight cultural environments.

By testing this model, this research aims to make several key contributions. First, it introduces the culturally nuanced construct of enterprising spirit into the mainstream creativity literature. Second, it disentangles the unique mediating roles of a cognitive belief (growth mindset) and a personality tendency (creative tendency). Third, it integrates a macro-cultural factor (cultural tightness) into a micro-psychological model, addressing calls for more contextually sensitive research on creativity (Chua et al., 2015; Karami et al., 2024). Ultimately, this study provides a more holistic understanding of how personal drive is transformed into creativity within specific cultural contexts.

## 3 Methods

### 3.1 Participants and procedure

A total of 600 undergraduate students were recruited from Qufu Normal University in China using a convenience sampling method. The inclusion criteria required participants to be full-time undergraduates enrolled at the university. After excluding questionnaires with omitted answers or patterned responses, 522 valid responses were retained, resulting in a validity rate of 90.40%.

The sample had a mean age of 18.26 years (*SD* = 0.63), and consisted of 213 males (40.8%) and 309 females (59.2%). In terms of academic discipline, participants were distributed as follows: 35.2% from Social Sciences, 28.5% from Natural Sciences, 21.6% from Arts and Humanities, and 14.7% from other or undeclared majors.

Data were collected through an online survey platform (Questionnaire Star). Participants received detailed instructions emphasizing the voluntary nature of participation, the confidentiality of their responses, and the academic use of the data. Informed consent was obtained from all participants before proceeding. The survey took approximately 15 min to complete. Upon submission, all participants received a small monetary compensation as a token of appreciation.

### 3.2 Measures

All scales were administered in Chinese. Back-translation was performed to ensure conceptual equivalence for scales originally in English. Confirmatory Factor Analysis (CFA) was conducted for each scale to assess structural validity. Model fit indices, including *χ*^2^/df, Comparative Fit Index (CFI), Tucker-Lewis Index (TLI), Root Mean Square Error of Approximation (RMSEA), and Standardized Root Mean Square Residual (SRMR), are reported below to support the construct validity of the instruments.

#### 3.2.1 Enterprising spirit

Enterprising spirit was measured using a 20-item scale developed by Kou (2011). Participants rated items on a 5-point Likert scale (1 = very poorly conformed, 5 = very well conformed). The scale encompasses four dimensions: purposefulness, conscientiousness, resilience, and desire for self-actualization, with higher scores indicating a higher level of enterprising spirit. In this study, Cronbach’s *α* was 0.89. CFA results indicated good fit: *χ*^2^/df = 2.58, CFI = 0.94, TLI = 0.92, RMSEA = 0.06, SRMR = 0.05.

#### 3.2.2 Creativity

Creativity was assessed using the 6-item scale developed by Madjar et al. (2011), which measures two dimensions: incremental creativity (3 items) and radical creativity (3 items). Responses were recorded on a 5-point Likert scale. Higher scores indicate higher levels of creativity. Cronbach’s *α* for the total scale was 0.87. The CFA showed acceptable fit: *χ*^2^/df = 3.21, CFI = 0.92, TLI = 0.90, RMSEA = 0.07, SRMR = 0.06.

#### 3.2.3 Creative tendency

The Chinese version of the Williams Creative Tendency Scale was used to measure creative personality (Luo and Lin, 2006; Nie and Zheng, 2005). This 16-item scale assesses traits such as adventurousness, curiosity, imagination, and challenge on a 5-point Likert format. Cronbach’s *α* was 0.77 in this study. CFA supported the four-factor structure: *χ*^2^/df = 2.89, CFI = 0.91, TLI = 0.90, RMSEA = 0.06, SRMR = 0.07.

#### 3.2.4 Cultural tightness

Cultural tightness was measured using the 6-item scale adapted from Gelfand et al. (2011). Participants responded on a 5-point Likert scale, with higher scores indicating a stronger perception of a tight cultural environment. Cronbach’s *α* was 0.82. CFA indices were satisfactory: *χ*^2^/df = 3.05, CFI = 0.93, TLI = 0.91, RMSEA = 0.07, SRMR = 0.06.

#### 3.2.5 Growth mindset

The Growth mindset scale by Dweck (2006) was used. The 6-item scale includes three items measuring an entity theory and three measuring an incremental theory, rated on a 5-point Likert scale (1 = strongly disagree, 5 = strongly agree). After reverse-scoring the entity theory items, higher total scores indicate a stronger growth mindset. Cronbach’s *α* was 0.87. The CFA demonstrated good fit: *χ*^2^/df = 2.75, CFI = 0.95, TLI = 0.93, RMSEA = 0.06, SRMR = 0.05.

### 3.3 Data analysis strategy

Data analysis was performed using SPSS 26.0, the PROCESS macro (Version 4.0; Hayes, 2017), and R software (Version 4.3.1). First, descriptive statistics and Pearson correlations were computed for all main variables.

To test the hypothesized moderated mediation model, we followed the analytical procedures outlined by Hayes (2017). The mediation effects (*H2* and *H3*) of growth mindset and creative tendency were tested using PROCESS Model 4 with 5,000 bootstrap samples to generate bias-corrected 95% confidence intervals (CI). The moderation effect (*H4*) of cultural tightness on the path from creative tendency to creativity was tested using PROCESS Model 7. The Johnson-Neyman technique was applied to probe significant interaction effects.

Furthermore, to explore the complex interrelationships among all variables beyond the regression-based models, a correlation network analysis was conducted using the qgraph package in R. This analysis provides a visual representation of the partial correlations between variables, helping to identify central nodes and unique pairwise associations in the data network.

Prior to these analyses, all statistical assumptions were checked. Multicollinearity was assessed using Variance Inflation Factors (VIFs), with all values below 3 indicating no severe multicollinearity. Normality of residuals was inspected via Q–Q plots and the Shapiro–Wilk test, and homoscedasticity was confirmed using the Breusch-Pagan test.

## 4 Results

### 4.1 Preliminary analyses

#### 4.1.1 Common method Bias

Given that all variables were measured through self-report, we assessed the potential for common method bias using both procedural and statistical controls. Statistically, Harman’s single-factor test was conducted using SPSS 26.0. The results indicated that 12 factors had eigenvalues greater than 1, with the first factor accounting for 19.90% of the variance, which is below the 40% threshold, suggesting that common method bias was not a serious concern in this study.

#### 4.1.2 Descriptive statistics and correlations

Means, standard deviations, Cronbach’s alphas, and Pearson correlations among all study variables are displayed in Table 1. Age was not significantly associated with any substantive variable. Gender was dummy-coded (1 = male, 2 = female); males reported slightly higher creativity (*t* = 3.79, *p* < 0.001) than females.

**Table 1:** Means, standard deviations, and Pearson correlations among study variables.

Variable	Mean	SD	Enterprising spirit	Creative tendency	Cultural tightness	Growth mindset	Creativity
1. Age	18.26	0.63					
2. Gender	1.59	0.49	*0.95*	*0.27*	*1.28*	*−0.04*	3.788***
3. Enterprising spirit	3.60	0.60	**(0.89)**				
4. Creative tendency	3.53	0.52	0.33***	**(0.77)**			
5. Cultural tightness	3.79	0.66	0.36***	0.27***	**(0.82)**		
6. Growth mindset	3.27	0.84	0.29***	0.06	0.08	**(0.85)**	
7. Creativity	3.19	0.83	0.47***	0.40***	0.23***	0.31***	**(0.87)**

***p < 0.001 (two-tailed). The italicized line represents the *t*-value. Bold represents internal consistency coefficient. Cronbach’s alpha reliability coefficients for each multi-item scale are presented in parentheses on the diagonal.

More importantly, entrepreneurial spirit was positively correlated with creative tendency (*r* = 0.33, *p* < 0.001), cultural tightness (*r* = 0.36, *p* < 0.001), growth mindset (*r* = 0.29, *p* < 0.001), and creativity (*r* = 0.47, *p* < 0.001). Creative tendency showed significant positive associations with cultural tightness (*r* = 0.27, *p* < 0.001), growth mindset (*r* = 0.06, ns), and creativity (*r* = 0.40, *p* < 0.001). Cultural tightness was positively related to growth mindset (*r* = 0.08, ns) and creativity (*r* = 0.23, *p* < 0.001). Finally, growth mindset was positively correlated with creativity (*r* = 0.31, *p* < 0.001). Overall, the correlation patterns provided initial support for the hypothesized links among entrepreneurial spirit, creative tendency, cultural tightness, growth mindset, and creativity.

### 4.2 Correlation network analysis

**Figure 2:**
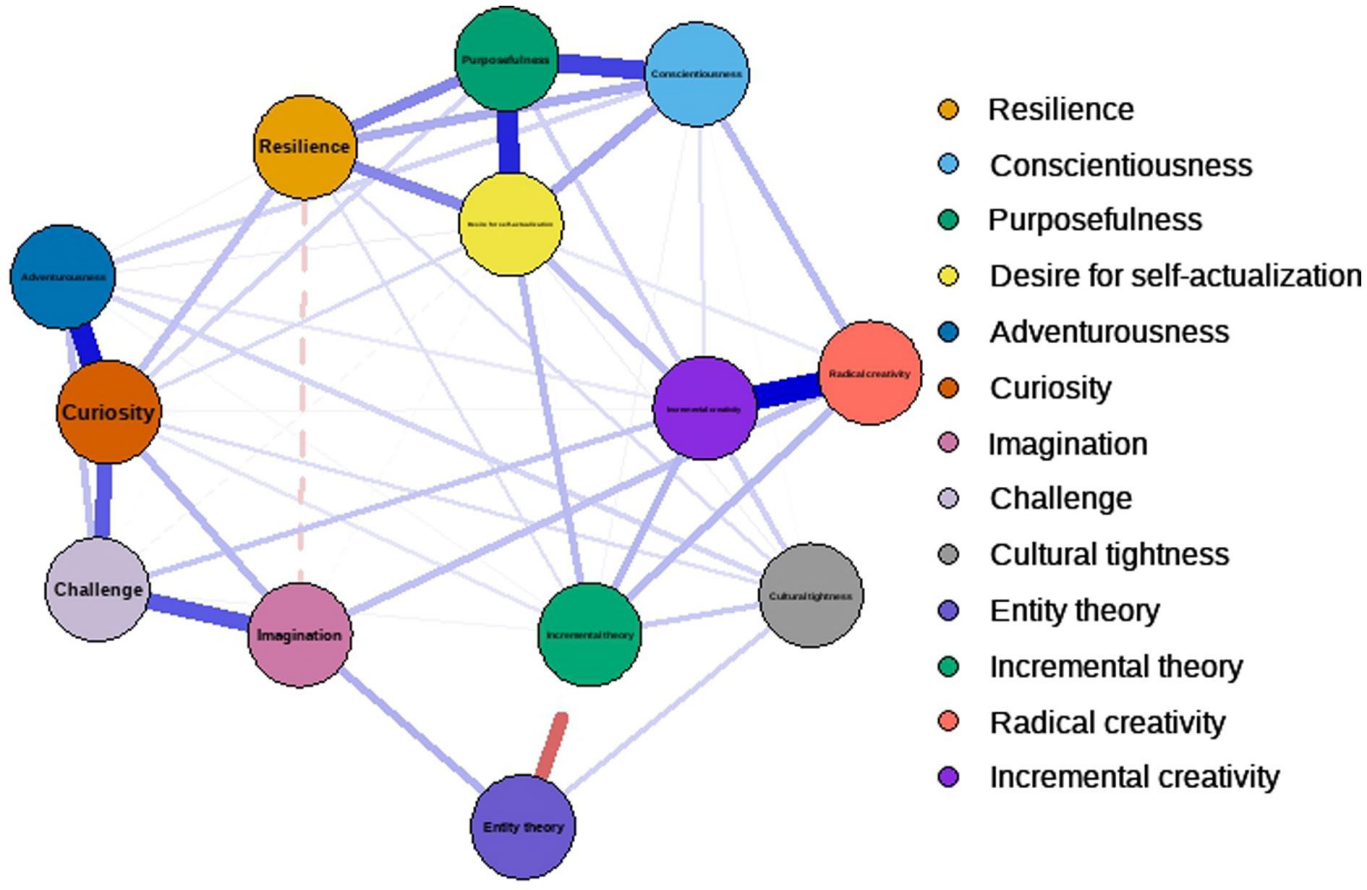
Results of correlation network analysis.

A partial correlation network was estimated using the qgraph package in R to explore the unique relationships between all variables after controlling for all others (Figure 2). The network revealed multiple moderate direct connections between variables across constructs. Notably, radical creativity showed direct edges with conscientiousness, incremental theory, and imagination. Incremental creativity was directly connected to desire for self-actualization, incremental theory, challenge, and cultural tightness. A negative edge was observed between incremental theory and entity theory, as expected.

**Figure 3:**
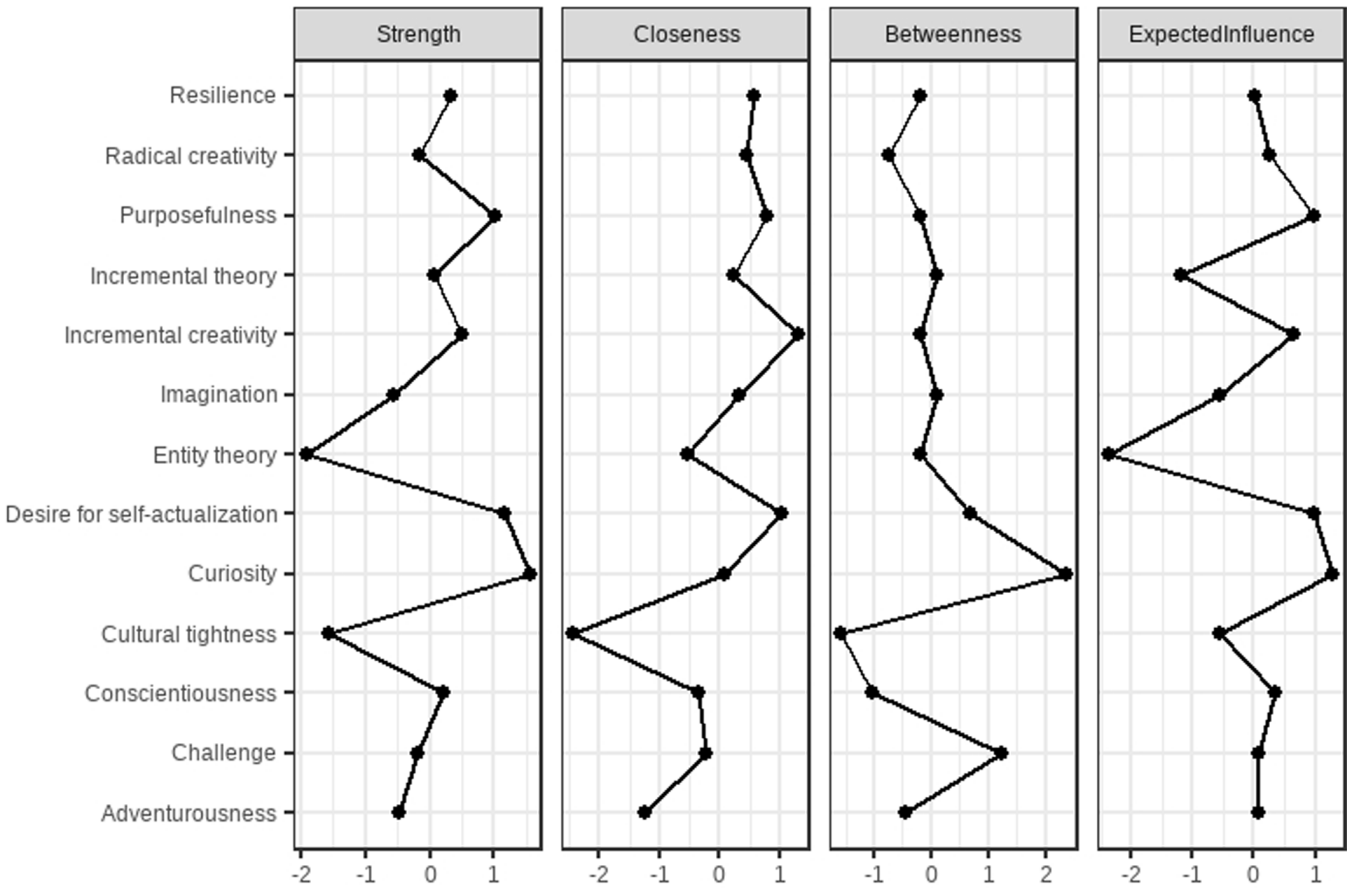
Centralized measurement of network nodes.

**Figure 4:**
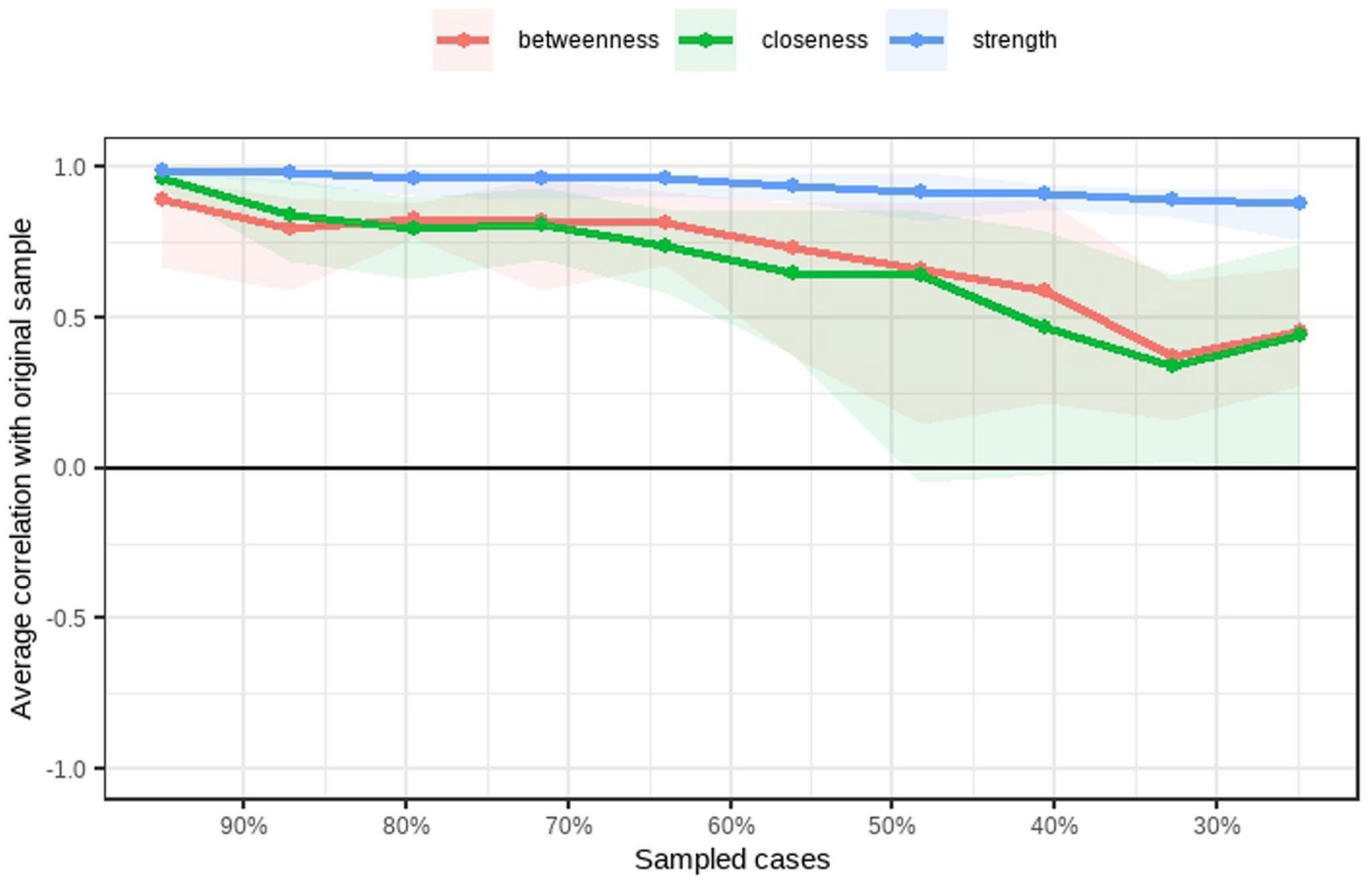
Stability testing of network centrality measures.

Centrality indices (strength, betweenness, and closeness) are plotted in Figure 3. Curiosity exhibited the highest strength centrality, indicating it had the strongest overall connections in the network, followed by desire for self-actualization and purposefulness. Curiosity also showed the highest betweenness centrality, suggesting it acts as a key bridge between other nodes. Incremental creativity had the highest closeness centrality, indicating it was most indirectly connected to all other variables in the network. The stability of these centrality indices was assessed using a case-dropping bootstrap procedure (Figure 4), which indicated that strength centrality was the most stable metric, consistent with recommendations in the network psychometrics literature (Epskamp et al., 2018).

### 4.3 Moderated mediation analysis

The hypothesized moderated mediation model was tested using Model 7 of the PROCESS macro for SPSS (Hayes, 2017) with 5,000 bootstrap samples. Gender and age were included as covariates. All continuous predictors were mean-centered prior to analysis.

#### 4.3.1 Direct and mediation effects

Enterprising spirit significantly predicted both mediators: growth mindset (*a*₁ = 0.12, *SE* = 0.03, *p* < 0.001) and creative tendency (*a*₂ = 0.19, *SE* = 0.03, *p* < 0.001). When both mediators and enterprising spirit were included in the model, growth mindset (*b*₁ = 0.18, SE = 0.03, *p* < 0.001) and creative tendency (*b*₂ = 0.17, *SE* = 0.03, *p* < 0.001) significantly predicted creativity. The direct effect of enterprising spirit on creativity remained significant (*c’* = 0.13, *SE* = 0.03, *p* < 0.001).

The bootstrap results indicated that the indirect effect of enterprising spirit on creativity through growth mindset was significant [*ab*₁ = 0.02, *BootSE* = 0.01, 95% *BootCI* (0.01, 0.04)]. Similarly, the indirect effect through creative tendency was also significant [*ab*₂ = 0.03, *BootSE* = 0.01, 95% BootCI (0.02, 0.05)]. Thus, *H1, H2, and H3 were supported*.

#### 4.3.2 Moderating effect of cultural tightness

**Figure 5:**
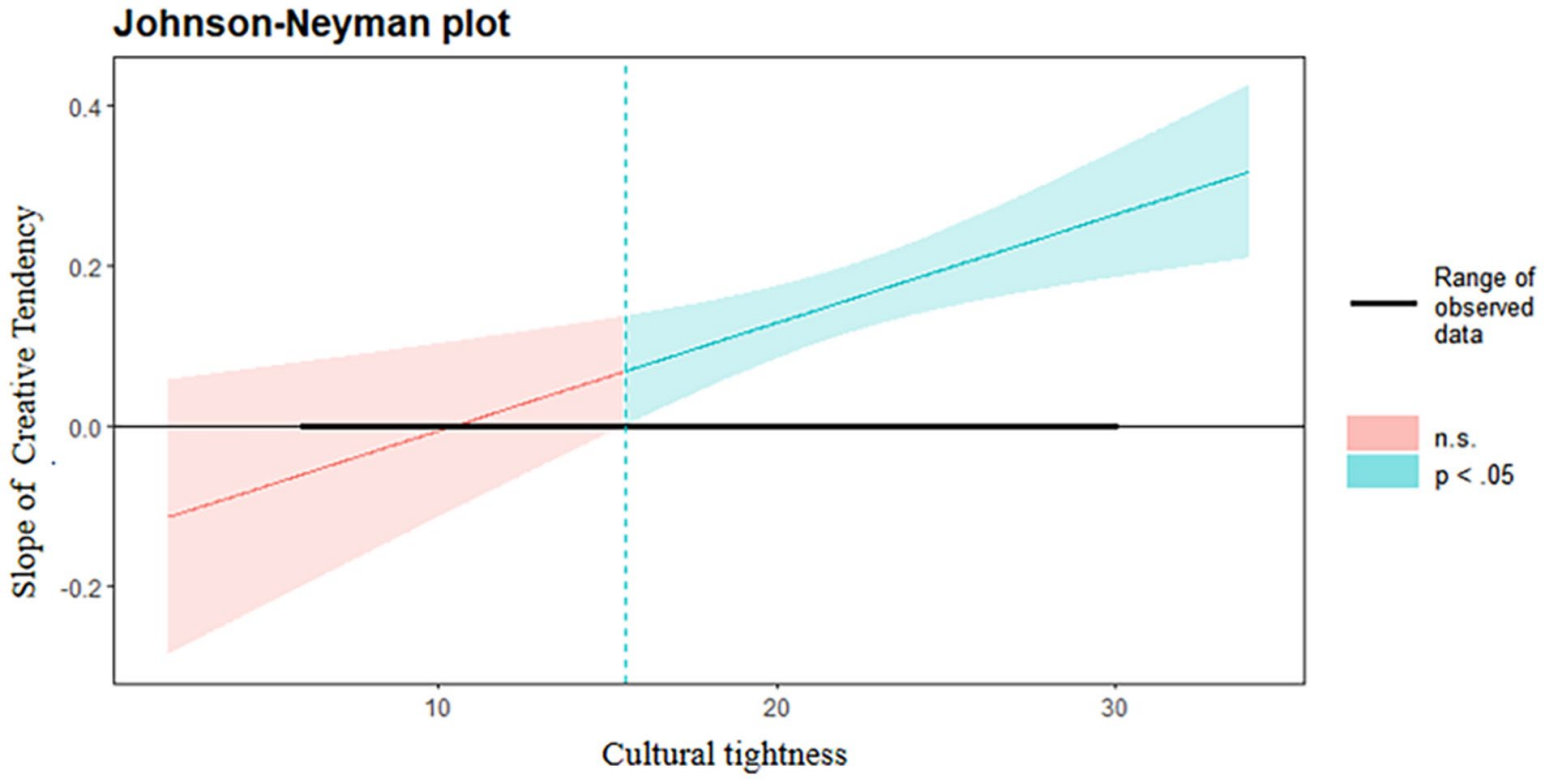
Moderating effect of cultural tightness on the relationship between creative tendency and creativity. The horizontal coordinate is cultural tightness (moderating variable), the vertical coordinate is the change in slope (change in regression coefficients in the regression equation with creativity as the dependent variable, creative tendency as the independent variable, and cultural tightness as the moderating variable), the blue area is the region of significance, and the red area is the region of non-significance.

As hypothesized, the interaction between creative tendency and cultural tightness on creativity was significant (*b* = 0.13, *SE* = 0.04, *p* = 0.001). The Johnson-Neyman technique was used to probe this interaction across the range of cultural tightness scores. As shown in Figure 5, the conditional effect of creative tendency on creativity was significant and positive across all observed values of cultural tightness, but became stronger as cultural tightness increased. *H4 was therefore supported*.

The moderated mediation index for the path via creative tendency was significant [*Index* = 0.03, *BootSE* = 0.01, 95% *BootCI* (0.01, 0.05)], confirming that the strength of this indirect effect was dependent on the level of cultural tightness (see Table 2).

**Table 2:** Results of the moderated mediation analysis (PROCESS Model 7).

Predictor	Creativity *β* [95% CI]	Growth mindset *β* [95% CI]	Creative tendency *β* [95% CI]
Gender	−1.43^***^ [−1.82, −1.04]	0.16 [−0.11, 0.43]	0.02 [−0.27, 0.31]
Age	0.17 [−0.15, 0.49]	0.21 [−0.02, 0.46]	−0.09 [−0.16, 0.31]
Enterprising spirit	0.13^***^ [0.09, 0.17]	0.12^***^ [0.08, 0.16]	0.19^***^ [0.15, 0.23]
Cultural tightness	0.02 [−0.03, 0.07]		
Growth mindset	0.18^***^ [0.12, 0.24]		
Creative tendency	0.17^***^ [0.11, 0.23]		
Creative tendency × Cultural tightness	0.13^**^ [0.05, 0.21]		
*R^2^*	0.36	0.08	0.14
*F*	41.25^***^	12.18^***^	24.93^***^

^**^p < 0.01, ^***^p < 0.001. Unstandardized coefficients are reported. CI, confidence interval.

## 5 Discussion

The present study proposed and tested a moderated mediation model examining how enterprising spirit influences creativity among college students, through the mediators of growth mindset and creative tendency, and under the moderating effect of cultural tightness. The results largely supported our hypotheses and offer meaningful contributions to the literature on creativity and positive psychology.

First, consistent with *H1*, enterprising spirit significantly predicted both incremental and radical creativity. This finding extends previous work linking achievement motivation to creativity (Chen et al., 2020) by introducing enterprising spirit—a construct deeply embedded in Confucian values yet aligned with global positive psychological assets—as a significant motivational antecedent of creative performance. This supports the notion that goal-directed resilience and proactive striving are essential for transforming creative potential into tangible outcomes.

Second, supporting *H2* and *H3*, both growth mindset and creative tendency mediated the relationship between enterprising spirit and creativity. This suggests two distinct psychological pathways through which enterprising spirit operates: a cognitive-belief pathway (growth mindset) and a personality-disposition pathway (creative tendency). The mediation through growth mindset aligns with Dweck’s (2006) theory, indicating that students with an enterprising spirit are more likely to believe in the developability of their abilities, thus embracing challenges and persisting through creative setbacks. The mediation through creative tendency underscores the role of adventurousness, curiosity, and intellectual openness—personality traits activated by enterprising motivation—that directly facilitate creative behavior (Guilford, 1950; Sternberg and Lubart, 1996).

Furthermore, the correlation network analysis offered a holistic view of variable interrelations. Curiosity emerged as the most central node, exhibiting the highest strength and betweenness centrality. This implies that curiosity functions as a critical hub connecting enterprising spirit, growth mindset, and creative tendencies, reinforcing its foundational role in creative cognition and motivation (Kashdan and Silvia, 2009). The strong negative correlation between entity and incremental theories further validates the bipolar structure of mindset beliefs.

Most notably, and in support of *H4*, cultural tightness moderated the second stage of the mediation via creative tendency. Specifically, the relationship between creative tendency and creativity was stronger in tight cultural environments. This finding resonates with Culture Fit Theory (Lu et al., 2024) and previous research suggesting that tight cultures provide structured environments and high collective efficacy that channel pre-existing creative tendencies toward socially endorsed innovations (Chua et al., 2015; Zhang, 2022). Contrary to some earlier findings that tightness may suppress radical creativity, our moderated mediation model reveals a more nuanced picture: tightness does not necessarily stifle creativity; rather, it amplifies the effectiveness of creative tendency in producing creative outcomes. This highlights the importance of considering cultural context as a potential enhancer rather than a barrier to creativity within certain pathways.

Interestingly, cultural tightness did not moderate the path through growth mindset. This suggests that the benefits of a growth mindset—an internal, self-reinforcing belief system—are relatively robust across cultural settings, supporting its universal utility in educational and innovation contexts.

## 6 Educational implications

These findings offer actionable insights for educators and policymakers aimed at fostering creativity in higher education:

### 6.1 Cultivating enterprising spirit

Educational programs should emphasize the development of enterprising qualities such as purposefulness, resilience, and courage through project-based learning, mentorship, and resilience-training workshops.

### 6.2 Promoting growth mindset

Interventions designed to strengthen students’ beliefs in the malleability of their abilities (e.g., through mindset workshops, reflective exercises) can enhance creative confidence and effort persistence.

### 6.3 Nurturing creative tendency

Curricula should encourage intellectual curiosity, risk-taking, and open-mindedness—for example, through interdisciplinary courses, innovation challenges, and safe-to-fail experimentation spaces.

### 6.4 Leveraging cultural context

In culturally tight environments, educators can frame creativity within collective goals and social relevance to align with normative expectations. In looser cultures, emphasizing individual exploration and autonomy may be more effective.

## 7 Conclusion

This study demonstrates that enterprising spirit enhances creativity among college students through both growth mindset and creative tendency, and that the latter pathway is moderated by cultural tightness. These findings advance our theoretical understanding of the psychological and cultural mechanisms driving creativity and provide a practical foundation for designing culturally sensitive interventions to nurture innovation and talent development in higher education.

Despite its insights, this study has several limitations. The cross-sectional design prevents causal inference. Future research should adopt longitudinal or experimental approaches to establish causality. Self-reported data may also introduce bias; incorporating behavioral or observational measures would strengthen validity. Additionally, the sample came from a single university, limiting generalizability—future studies should include more diverse cultural and academic contexts. Finally, cultural tightness was examined at the individual level; multilevel designs would better capture its collective nature.

These limitations point to meaningful future directions, including testing causal models over time, expanding measurement methods, and comparing cultural regions to enhance the external validity and practical relevance of the findings.

## Data Availability

The raw data supporting the conclusions of this article will be made available by the authors, without undue reservation.
